# The effect of haptic cues on motor and perceptual based implicit sequence learning

**DOI:** 10.3389/fnhum.2014.00130

**Published:** 2014-03-28

**Authors:** Dongwon Kim, Brandon J. Johnson, R. Brent Gillespie, Rachael D. Seidler

**Affiliations:** ^1^HaptiX Laboratory, Department of Mechanical Engineering, University of Michigan, Ann ArborMI, USA; ^2^Neuromotor Behavior Laboratory, Department of Psychology, School of Kinesiology, University of Michigan, Ann ArborMI, USA

**Keywords:** sequence learning, haptic cue, motor memory, serial reaction time task

## Abstract

We introduced haptic cues to the serial reaction time (SRT) sequence learning task alongside the standard visual cues to assess the relative contributions of visual and haptic stimuli to the formation of motor and perceptual memories. We used motorized keys to deliver brief pulse-like displacements to the resting fingers, expecting that the proximity and similarity of these cues to the subsequent response motor actions (finger-activated key-presses) would strengthen the motor memory trace in particular. We adopted the experimental protocol developed by Willingham (1999) to explore whether haptic cues contribute differently than visual cues to the balance of motor and perceptual learning. We found that sequence learning occurs with haptic stimuli as well as with visual stimuli and we found that irrespective of the stimuli (visual or haptic) the SRT task leads to a greater amount of motor learning than perceptual learning.

## INTRODUCTION

Motor learning, especially in its latter phases, often takes place without dedicated attention and without awareness for either the process or the content of what is learned. Fitts and Posner (1967) called this phase of motor learning *autonomous.* *Implicit learning* describes not just motor but any type of learning, and is similarly characterized by a lack of awareness for the process or the content of what is learned (Cleeremans, 1993; Reber, 1993). Implicit learning is often studied using the serial reaction time (SRT) task, which was introduced by Nissen and Bullemer (1987). In the SRT task, participants respond to stimuli presented in one of four locations by pressing a corresponding key. When a repeating sequence of stimuli structured according to certain rules is presented unbeknownst to participants, reaction times (RTs) and error rates decrease with practice. If the stimuli later appear randomly, the participants do not respond as quickly. Participants are often unaware of the existence of the structure and are unable to express the sequence, implying that learning occurred implicitly (Willingham et al., 1989).

Since the SRT task involves responding with a key press, a portion of RT or error rate improvements may be attributable to motor learning. That is, the sequence is learned in terms of a sequence of motor responses, a view termed R-R (response-to-response) learning. This view contends that the motor systems governing active movement generate a memory trace of successive motor actions. This allows participants to anticipate the next response at a given trial even without perceiving stimuli (Hoffmann and Sebald, 1996; Nattkemper and Prinz, 1997; Willingham, 1999). In this sense, R-R learning would presumably be a type of motor learning, while the alternative, S-S (stimulus-to-stimulus) learning would be a type of perceptual learning. S-S learning assumes that perceptual memory systems are involved in forming a representation for successive stimuli. Participants use representations to predict the next stimulus in a sequence based on associations with the previous stimulus even when they are not able to thoroughly recall the sequence (Fendrich et al., 1991; Howard et al., 1992).

A fair amount of attention has been dedicated to determining whether and how motor learning and perceptual learning contribute differently to obtaining sequence knowledge in the SRT task (Bischoff-Grethe et al., 2004; Gheysen et al., 2009; Nemeth et al., 2009; Hallgato et al., 2013). These studies have been performed with a variety of experimental paradigms in an attempt to disentangle the motor and perceptual contributions (e.g., Willingham, 1999; Remillard, 2003; Hoffmann et al., 2003). Most of these studies have, however, been undertaken exclusively using visual stimuli (e.g., Mayr, 1996; Willingham, 1999); only a few studies have explored sequence learning using other stimulus modalities (e.g., Zhuang et al., 1997; Abrahamse et al., 2008).

A stimulus modality of particular value in the development of motor skills is the haptic modality. Haptic cues invariably accompany motor actions that involve contact with objects in the environment. Even non-contact motor tasks involve proprioceptive, skin stretch, and inertial force cues. Especially for motor skills that involve sequenced actions, an accompanying sequence of haptic cues might be involved in the development and retention of motor skills. Such cues can signal the successful completion of a motor action. For example, the detent or click-feel and subsequent bedding of a key on a computer keyboard together signal completion of a keypress and possibly play a role in the development of chunked keying sequences.

A number of computer-assisted motor training environments based on haptic technology have been created, hoping to leverage the role of haptic stimuli in motor learning (Armstrong, 1970; Rosenberg, 1992; Gillespie et al., 1998; Feygin et al., 2002; O’Malley and Gupta, 2003; Tsutsui and Imanaka, 2003; Kahn et al., 2006; Liu et al., 2006; Grindlay, 2008; Marchal-Crespo and Reinkensmeyer, 2008; Lee and Choi, 2010). The idea is to synthesize the appropriate haptic cues using a motorized device and potentially to automate the role of another human who provides manual guidance. Results have been mixed in many studies, but a few studies have demonstrated the benefit of automated guidance for mastering motor skills, especially the studies of Feygin et al. (2002), Marchal-Crespo and Reinkensmeyer (2008), and Choi and Lo (2011).

Motivated by these studies, and hoping to introduce some rigor into research involving haptic devices for motor learning, we introduce haptic stimuli to the SRT task. In particular, we introduce haptic cues that produce motion in the fingers. By back-driving the tendons and joints of the fingers, which are associated with kinesthetic receptors, haptic stimuli could enhance motor memory based on their association with the excitation of similar kinesthetic cues that occur during the response key presses. Consequently, we expect that a SRT task using haptic stimuli could show enhanced motor-based learning relative to a SRT task using visual stimuli. The vibrotactile stimulus introduced in Abrahamse et al. (2008) could also be regarded as a haptic stimulus, but was different in that cues of small amplitude, high frequency (200 Hz) vibration were presented to the skin on the proximal phalanx of a finger. Note that the haptic stimuli developed in this study trigger significant movement of the tendons, muscles, and joints in addition to the skin of the fingers.

In this study, our main objective was to investigate how haptic cues contribute to the balance of motor and perceptual learning. We adopted the SRT protocol developed by Willingham (1999) to distinguish motor and perceptual components of learning, adding haptic cues alongside the standard visual cues. Many variants of the SRT task have been developed to eliminate certain effects and to even more finely distinguish types of motor learning. In particular, the learning of the correct answer button sequence in the egocentric space (response-based learning) can be distinguished from learning of the finger movement patterns (effector-based learning; e.g., Willingham et al., 2000). Willingham (1999), Willingham et al. (2000), and Witt and Willingham (2006) showed that sequence knowledge can pertain to sequences of response location, whereas Park and Shea (2005), Bapi et al. (2000), Verwey and Wright (2004), and Verwey and Clegg (2005) demonstrated the existence of an effector-specific component in sequence learning. However, Deroost et al. (2006) and Berner and Hoffmann (2009) suggested the contributions of effector-independent (response location) and effector-specific acquisitions to learning can coexist. The contribution of eye movements, termed oculomotor learning, is another type of motor learning. Gheysen et al. (2009) and Nemeth et al. (2009) devised modified versions of Willingham’s SRT design by presenting stimuli at only one location to eliminate the participation of oculomotor learning. In the present study we do not attempt to distinguish effector specific learning and response location learning. We also did not attempt to eliminate oculomotor learning as the co-location of stimuli in the haptic case would involve the coding (and de-coding by our participants) of information in time rather than space. We are interested in a presentation of haptic stimuli that is most natural and ecologically meaningful in the context of the button-pressing task.

In Willingham’s protocol, participants performed a SRT task in an incompatible mapping for the training phase and in a compatible mapping for the transfer phase. In the incompatible mapping, participants were asked to press one key to the right of the stimulus (shifted right). If the stimulus was on the far right, participants pressed the key on the far left (wrapping around). In the compatible mapping, there was a direct spatial correspondence between each stimulus and key. To see whether participants’ learning was oriented to stimulus-based or response-based sequences, his study investigated transfer to the compatible mapping (without shift) in the following two conditions. In the perceptual condition, the transfer stimulus sequence was identical to that used during training [but participants would press different keys due to the change from incompatible (shifted) to compatible (unshifted) mapping]. Whereas in the motor condition, the stimulus sequence was shifted left so that response sequences were identical to those in the training phase. Willingham (1999) reported better transfer in the motor group compared to the perceptual group, suggesting that participants learn sequences at the motor response level. In contrast to the Willingham study, which investigated whether perceptual or motor learning is predominant in visual stimuli, the present study focuses on determining whether one modality (haptics or vision) would better facilitate motor learning than perceptual learning. We expected that the haptic modality is more closely tied to the motor system and would thus favor motor learning.

## MATERIALS AND METHODS

### PARTICIPANTS

A total of 32 volunteers (24 male), from the University of Michigan, ranging in age from 19 to 33 years (24.28 ± 3.77SD), participated in the study. All participants reported normal or corrected-to-normal vision, and no neurological or motor deficit. None of the participants had previous experience with SRT tasks and were not made aware of our hypotheses. All gave written informed consent. The experiment was approved under the University of Michigan’s Behavioral Sciences Institutional Review Board.

### APPARATUS

**FIGURE 1:**
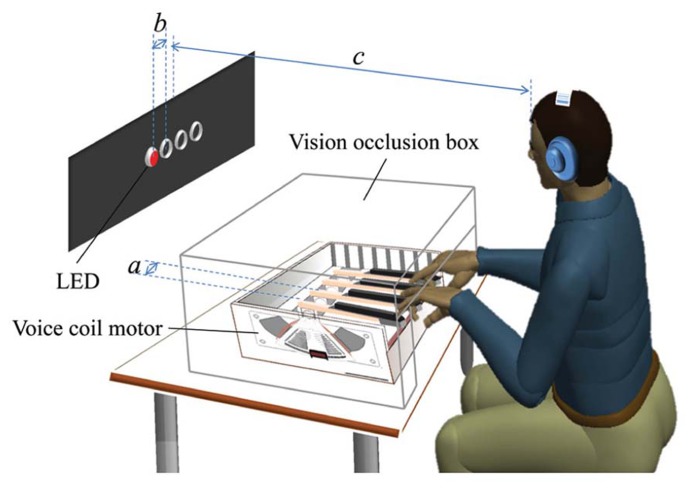
**A schematic diagram of the experimental apparatus and setup.** Four lever-shaped keys were motorized using flat voice coil motors (VCMs) and placed under computer control. The keys were spaced 6.5 cm centerline to centerline (dimension *a*). Visual stimuli were presented by the lighting of four horizontally positioned red LEDs spaced 5 cm apart (dimension *b*) and haptic stimuli were provided by the injection of an upward half-sinusoid pulse in the reference position of the key. A participant sat and rested her/his ring and index fingers of both hands on the keys. The center of the four LEDs was about 90 cm away from the participant’s eyes (dimension *c*). She/he was allowed to adjust the height of the chair for comfort and the participant’s view of her/his hands was occluded using a box. White noise was presented through headphones.

We fabricated a custom keyboard of four motorized keys using a custom flat voice coil motor on each key to present the haptic cues (see **Figure 1**). The four lever-shaped keys were instrumented with optical encoders for position-control and to record participants’ responses. The four keys were controlled by a 2.27 GHz personal computer running Windows 7. The keys were equally spaced, and the distance between key centers was 65 mm (dimension *a *in **Figure 1**). To indicate to participants when their responses had been registered by the computer, the mechanical behavior of each key included a detent or click-feel. In particular, the characteristic feel of a buckle spring keyswitch was adopted. This type of keyswitch cues the user that their key press has been registered or captured, and is preferred in commercially available keyboards because it supports fast typing speeds and low error rates (Brunner and Richardson, 1984). See Kim et al. (2013) for further implementation details.

**FIGURE 2:**
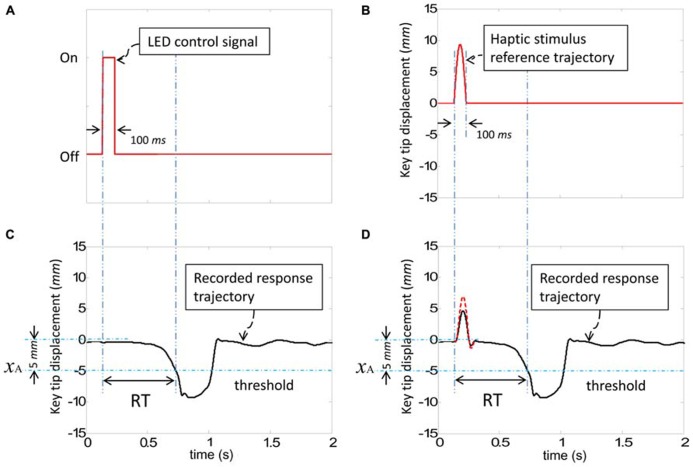
**The command signals for visual and haptic stimuli and recorded responses.** **(A)** The control signal to turn on/off the LED for visual stimuli. **(B)** The reference position to be followed by the tip of the key for haptic stimuli. **(C)** A sample of a typical recorded trajectory responding to a visual stimulus. **(D)** A sample of typical recorded trajectories in response to a haptic stimulus (dashed line: without the finger resting on the key, solid line: with the finger resting on the key). Reaction time (RT) was defined as the difference between the initial stimulus command and the point at which the key crosses the threshold. Both visual and haptic stimuli were presented for a 100 ms time interval.

Stimuli to elicit participants’ responses in the SRT task were presented in two ways: visual and haptic. Visual stimuli were provided by the sequential lighting of four horizontally positioned red LEDs spaced 5 cm apart (dimension *b *in **Figure 1**) against a black background situated about 90 cm from the participant’s eyes (dimension *c *in **Figure 1**, visual angle: 9.5°). Haptic stimuli were generated by injecting an upward 100 ms half-sinusoid pulse in the reference position of the key, with an amplitude of 7 mm with respect to the tip of the key [see Kim et al. (2013) for details], as shown in **Figure 2**. A corresponding pulse in force and an associated pulse excursion in position would be delivered to a finger.

The haptic stimuli were delivered to the index and ring fingers of both hands resting on the keys. Each finger was mapped to a key (labeled 1–4 from left to right) with the left ring finger responding on key 1, left index finger on key 2 and so on, as illustrated in **Figure 1**. We followed Abrahamse et al. (2008) in presenting stimuli to the index and ring fingers instead of the adjacent fingers in order to enhance the ability to discriminate which finger was being presented with a haptic cue.

Reaction time (RT) was defined as the difference between the initial stimulus command and the time at which the threshold *x*_A_ was crossed, as presented in **Figure 2**. The participants’ view of their hands was blocked using a box and white noise was presented via headphones in order to eliminate spurious audio cues.

### PROCEDURE

All participants were randomly and evenly divided into two groups: one group (*N *= 16) responded to visual stimuli (the visual group), while the other group (*N *= 16) responded to haptic stimuli (the haptic group). Each of these two groups was randomly and evenly divided into two subgroups. One of the two subgroups (*N *= 8) was assigned to the transfer condition that preserved stimulus sequence across transfer (perceptual condition), whereas the other subgroup (*N *= 8) was assigned to the transfer condition that preserved response sequence across transfer (motor condition). For convenience, the four subgroups will be named *visual-perceptual*, *visual-motor*, *haptic-perceptual, and haptic-motor* subgroups, respectively. The motor and perceptual conditions will be further explained below. The motor and perceptual conditions were different only in name up until the transfer phase, at which point different sequences (one of them shifted) were presented to the two subgroups.

Responses to stimuli were made either in the so-called “incompatible” or “compatible” stimulus-response mappings. In the incompatible mapping, participants were instructed to press the key one position to the right of the position at which the stimulus appeared. If the stimulus on the far right appeared, they were to press the key on the far left.

The experiments unfolded in three stages: the familiarization, training, and transfer phases. Before the SRT task began, the familiarization phase was introduced to allow participants to practice making responses on the keyboard apparatus. This phase provided an opportunity to teach participants how to press the keys properly and to ensure they understood the incompatible and compatible stimulus-response mappings. Participants were allowed to view their hands during this exercise to facilitate this learning process. When participants were able to demonstrate proper key pressing and stimulus-response mappings, the familiarization phase was stopped, and the SRT task began.

In the training phase, all participants performed the SRT task in the incompatible stimulus-response mapping, regardless of whether visual or haptic stimuli were presented. All four subgroups experienced the same stimulus sequences during this phase. Shortly afterward, the transfer phase followed, to test whether or not the sequence knowledge acquired during the training phase appeared in the compatible mapping and whether it appeared to differing degree according to the stimulus type (haptic/visual) or presentation mode (perceptual/motor).

Now we define the perceptual and motor presentation modes after transfer in detail. In the transfer phase, the *visual-perceptual* and the *haptic-perceptual* subgroups responded using the compatible mapping to cues delivered in sequences that were not altered from those delivered during the training phase (under the perceptual condition). However, the *visual-motor* and *haptic-motor* subgroups responded during transfer using the compatible mapping to cues delivered in sequences that were shifted so that the sequence of *responses* (key presses) would turn out identical to those made in the training phase. That is, in the perceptual condition, the sequence of responses was *shifted* since cue delivery (perceived sequences) was the same across the training and transfer phases, while under the motor condition, the sequence of *responses* *remained consistent* (*unshifted*, with motor responses the same across training and transfer phases), since cue delivery was shifted across the training and transfer phases.

Both visual and haptic stimuli were presented for a 100 ms time interval and then turned off, as described in **Figure 2**. The response-to-stimulus interval (RSI) was 250 ms for correct responses. All participants were asked to respond as fast as possible without making errors in a manner that corresponded to the incompatible mapping in the training phase and compatible mapping in the transfer phase. Responses were declared erroneous when participants failed to press the appropriate key or make a response within 1.5 s of stimulus presentation. Errors were signaled to participants via audio tones and an extended RSI of 1 s. Thirty second breaks were provided between blocks.

Stimulus events (cues) were organized into sequences of 12 and were constructed according to the rules of second-order conditional (SOC) sequences. In a SOC sequence, each event can be predicted only by a unique combination of two preceding events and each pairwise association is equally likely so that pairwise association cannot be used to predict subsequent stimuli (Reed and Johnson, 1994). These sequences were then organized into blocks of 108 events. Two types of blocks were presented: sequence blocks that consisted of one SOC (242134123143) repeated nine times, and pseudorandom blocks which consisted of nine distinct, successively presented SOCs picked from a pool of 12. Sequences were presented seamlessly such that participants were only aware of a set of 108 events, and each SOC sequence presented was initiated at the beginning.

The training phase comprised one pseudorandom block succeeded by seven sequence blocks, a pseudorandom block (block 9) and a final sequence block (block 10). The first pseudorandom block acclimated participants with the task and established a baseline RT while the final pseudorandom block allowed us to differentiate sequence learning from general practice effects. Transfer consisted of two pseudorandom blocks (blocks 11 and 12) for adjusting to the new mapping; one sequence block (block 13) and a final pseudorandom block (block 14). A given participant never experienced the same pseudorandom block twice. Median RT and error percentage were displayed for participants between blocks.

### AWARENESS SURVEY

After the experiment, a 6-question survey was conducted to determine how much explicit knowledge participants had gained. Question 1 asked participants to choose from a list of four alternatives the statement that best described the task carried out: (1) “Stimulus presentation was completely random,” (2) “Some fingers had to respond more often than others,” (3) “Sometimes I wanted to respond before stimulus presentation,” and (4) “Stimulus presentation was mostly structured” (Abrahamse et al., 2008). Questions 2 and 3 were adapted from the process dissociation procedure (PDP) introduced by Destrebecqz and Cleeremans (2001). Question 2 required participants to generate the 12-event sequence experienced (inclusion) while Question 3 asked participants to generate another 12-event sequence that completely avoided the first (exclusion). Participants were told to recall the sequence as experienced in the training phase (Bischoff-Grethe et al., 2004). During these exercises, populating the sequence with repeating smaller patterns was not allowed (e.g., 123412341234 would not be a valid response). Therefore chance level was 0.33. In Question 4, six different SOC sequences were presented through the apparatus in whichever modality participants trained and the participants were asked to identify the correct one (the sequence experienced during training). Questions 5 and 6 asked the participants to rank their engagement in the task and the task’s difficulty on a scale of 1 to 5 (5 being “very engaged” and “very difficult,” respectively).

### TRAINING SCORES, TRANSFER SCORES, AND AWARENESS SCORE

Median RTs were obtained for each participant and block of data (nominally 108 responses), though RTs from erroneous responses and trials immediately following erroneous responses were excluded. We chose median RT over mean RT for its robustness to outliers. The median was then averaged across the participants within a group (subgroup) to determine an overall RT value for each block and group (subgroup). Two learning scores were computed in terms of RT: a Training RT Score and a Transfer RT Score. The Training RT Score was determined by subtracting the average of the RT values in blocks 8 and 10 (sequence blocks) from the RT value in block 9 (a random block). The Transfer RT Score was determined by subtracting the RT value in block 13 (a sequence block) from the average of the RT values in blocks 12 and 14 (random blocks).

For Error Scores, error rates were first obtained for each participant and block of data. We then averaged them across the participants within a group (subgroup) to determine an overall error value for each block and group (subgroup). Two Error Scores were computed: a Training Error Score and Transfer Error Score. The Training Error Score was determined by subtracting the average of the error rates in blocks 8 and 10 (sequence blocks) from the error rate in block 9 (a random block). The Transfer Error Score was determined by subtracting the error rate in block 13 (a sequence block) from the average of the error rates in blocks 12 and 14 (random blocks).

We computed Training RT Scores and Training Error Scores for each group (visual/haptic) but not condition (no subgroups) since there were no differences in the cues presented to the subgroups in the training phase.

To obtain Awareness Scores, the sequences generated by participants in response to Questions 2 and 3 were broken into 3-element chunks. The actual sequence used in that participant’s sequence blocks was likewise divided. Chunks from the generated sequences were compared against those in the actual sequence and the number of correct chunks was divided by 12 (the maximum possible number of correct chunks) resulting in an awareness score between zero and one. The awareness scores were calculated for each participant in both inclusion and exclusion recall tasks.

### DATA ANALYSIS

We employed ANOVA and *t*-tests for statistical analysis as elaborated below. Statistical analyses were performed with SPSS (Windows v.21, SPSS Inc.). Significance level was set at 0.05.

We used a mixed-design ANOVA and repeated-measure ANOVA to investigate performance change across repeated measurements and to assess how stimulus (visual/haptic) or/and condition (perceptual/motor) influenced these changes. Two-way ANOVAs were used to test for significant differences in mean across the four subgroups. If the sphericity assumption in ANOVAs was violated, Greenhouse–Geisser adjusted P values were used.

Independent-sample *t*-tests were carried out to test whether the two groups were significantly different in means. We used one-tailed *t*-tests to determine whether learning scores were greater than zero. Paired-sample *t*-tests were employed in cases where data were paired.

## RESULTS

**FIGURE 3:**
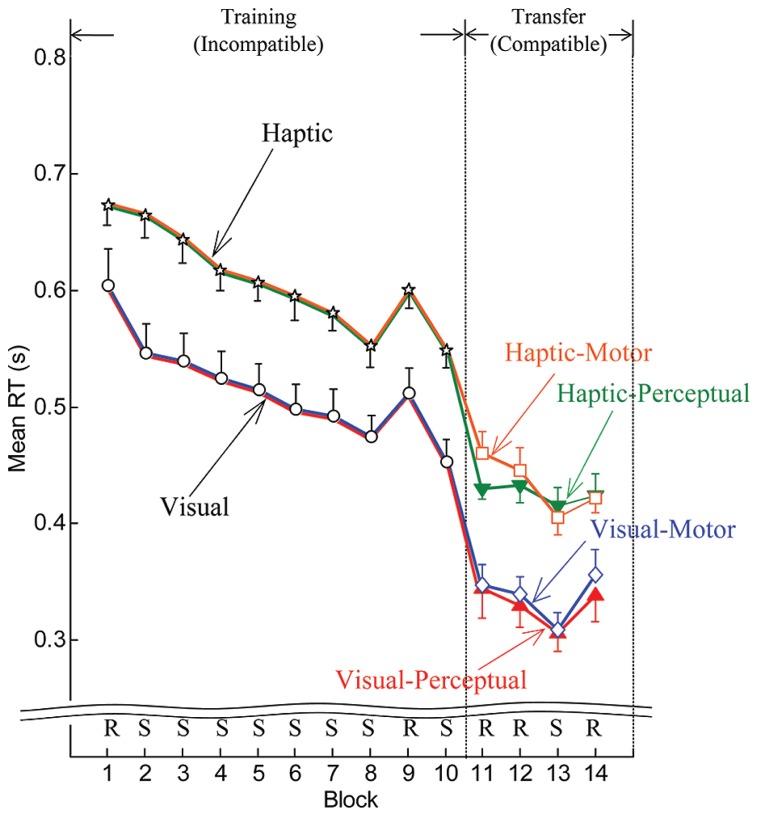
**Mean by group and subgroup of individual median RTs for the training phase (blocks 1–10) and transfer phase (blocks 11–14).** Error bars are ± 1 standard error of the mean. R and S stand for pseudorandom and sequenced stimuli, respectively.

Reaction times improved with practice regardless of whether the cues were delivered in the visual or the haptic modality. **Figure 3** shows the means of individual median RTs computed for both the visual and haptic groups in the training phase and for each of the four subgroups in the transfer phase. Downward trends in RT appeared as participants practiced in the training phase in blocks 2 through 8. Since a pseudorandom block followed sequence block 8, increases in RT occurred at block 9. But RTs decreased again for sequence block 10. Notable drops in RT occurred at the transition from the training phase to the transfer phase (between blocks 10 and 11), which could be expected since the compatible mapping is easier than the incompatible mapping. Also, as expected, decreases in RT appeared at sequence block 13, because participants benefited from knowledge of the sequence acquired during the training phase.

**FIGURE 4:**
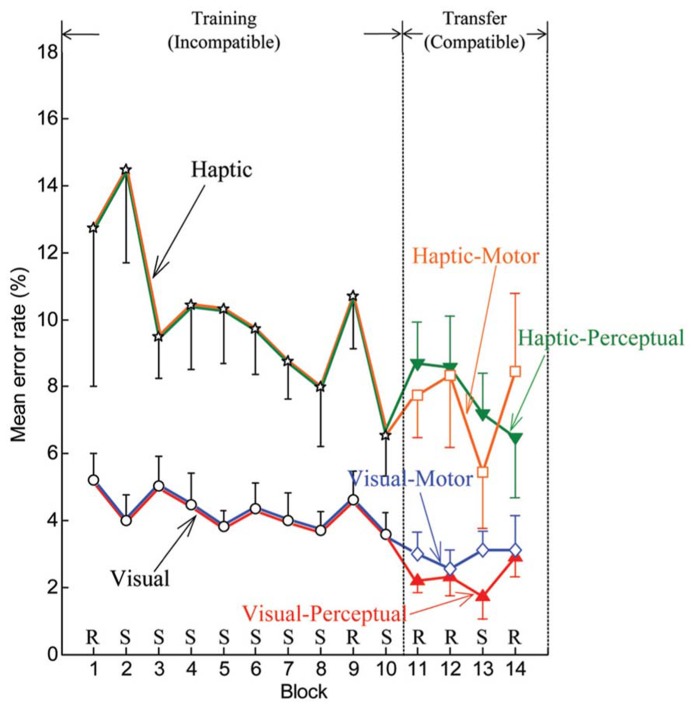
**Mean by group and subgroup of individual error rates for the training phase (blocks 1–10) and transfer phase (blocks 11–14).** Error bars are ± 1 standard error of the mean. R and S stand for pseudorandom and sequenced stimuli, respectively.

Error rates also improved with practice. **Figure 4** displays the means of individual error rates computed for both the visual and haptic groups in the training phase and for each of the four subgroups in the transfer phase. During blocks 2–8, downward or plateau trends in error rate appeared for the visual and haptic groups. As expected, increases in error rate were exhibited at pseudorandom block 9 in comparison with the surrounding blocks for each group. During the transfer phase, decreases in error rate occurred at sequence block 13 for all subgroups other than the visual-motor subgroup.

### TRENDS IN THE TRAINING PHASE (BLOCKS 2–8)

Because all subgroups experienced the same sequences in the training phase, we did not differentiate the groups by Condition; we partitioned participants into the visual and haptic groups regardless of the perceptual and motor conditions.

Repeated-measure ANOVAs were performed on median RTs and error rates from block 2 to block 8 to evaluate participants’ performance with Block (seven levels: blocks 2–8) as a within-subject variable and Stimulus (two levels: visual and haptic) as a between-subject variable. RTs for the haptic group were in general longer in comparison to the visual group (see **Figure 3**). ANOVA produced a significant main effect of Stimulus [*F*(1,30) = 12.463, *MSE *= 0.515, *p* < 0.001, $\eta_{p}^{2}$ = 0.294]. A main effect of Block [*F*(4.128,123.839) = 32.381, *MSE *= 0.048, *p* < 0.001, $\eta_{p}^{2}$ = 0.519] was also significant. Polynomial contrasts reported a linear trend in Block [*F*(1,30) = 104.388, *MSE *= 0.197, *p* < 0.001, $\eta_{p}^{2}$ = 0.777]. The other main and interaction effects were not significant (all *p* > 0.1). Likewise, error rates were generally higher in the haptic group than in the visual group regardless of Condition (see **Figure 4**). ANOVA reported a significant main effect of Stimulus (visual/haptic) [*F*(1,30) = 17.458, *MSE *= 1800.506, *p* < 0.001, $\eta_{p}^{2}$ = 0.368]. The other main and interaction effects did not reach significance (all *p* > 0.1).

### SEQUENCE-UNSPECIFIC LEARNING

We investigated sequence-unspecific learning in the visual and haptic groups in the training phase by comparing pseudorandom blocks 1 and 9. A repeated measures ANOVA was performed on RT with Block (two levels: blocks 1 and 9) as a within-subject variable and Stimulus (two levels: visual and haptic) as a between-subject variable. A main effect of Stimulus [*F*(1,30) = 8.291, *MSE *= 0.101, *p* < 0.01, $\eta_{p}^{2}$ = 0.217] reached significance, implying that the haptic group showed higher RTs at the pseudorandom blocks than the visual group. A main effect of Block [*F*(1,30) = 30.234, *MSE *= 0.110, *p* < 0.005, $\eta_{p}^{2}$ = 0.502] was significant, indicating that sequence-unspecific learning occurred in both the visual and haptic groups. The interaction (Stimulus × Block) was not significant (*p* = 0.533).

Similarly, a repeated measures ANOVA was used to analyze error rate with Block (two levels: blocks 1 and 9) as a within-subject variable and Stimulus (two levels: visual and haptic) as a between-subject variable. A main effect of Stimulus [*F*(1,30) = 7.431, *MSE* = 739.79, *p* < 0.05, $\eta_{p}^{2}$ = 0.751] was significant, indicating that the haptic group exhibited higher error rates at the pseudorandom blocks than the visual group. The ANOVA on error rate reported no other main or interaction effects (all *p* > 0.1).

### TRAINING RT SCORES AND TRAINING ERROR SCORES

**FIGURE 5:**
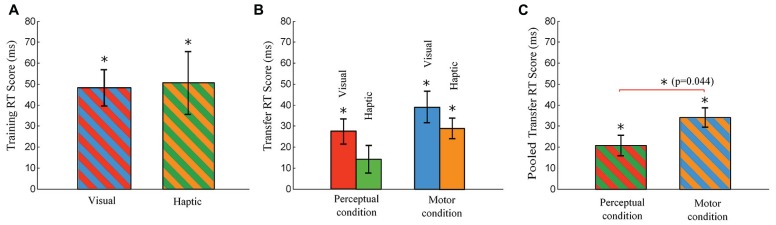
**Training RT Score and Transfer RT Score.** The Training RT Score is defined as a difference in RT between the means of blocks 8 and 10 and block 9, and the Transfer RT score is defined as a difference in RT between the means of blocks 12 and 14 and block 13. **(A)** Training RT Scores of the visual and haptic groups (no differences in the cues presented to the subgroups in the training phase). **(B)** Transfer RT Score of the visual-perceptual, haptic-perceptual, visual-motor, and haptic-motor subgroups. **(C)** Pooled Transfer RT Score (averaged across the visual and haptic groups) in each condition. The motor group shows a significantly greater pooled Transfer RT Score than the perceptual group (ANOVA, *p* = 0.044). Error bars are ± 1 standard error of the mean. An asterisk on a line linking bars indicates a significant difference between two groups (subgroups) while an asterisk above a bar indicates a significant difference from zero.

Each Training RT Score quantifies the increase in RT of pseudorandom block 9 over the average RT of sequence blocks 8 and 10. One-tailed *t*-tests revealed that the Training RT Scores were significantly greater than zero in the visual and haptic groups, as indicated with asterisks over the bars in **Figure 5A**. However, independent-sample *t*-tests comparing between groups reported that there was no significant difference between the visual and haptic groups in Training RT Scores [*t*(30) = 0.139, *p* = 0.89].

**FIGURE 6:**
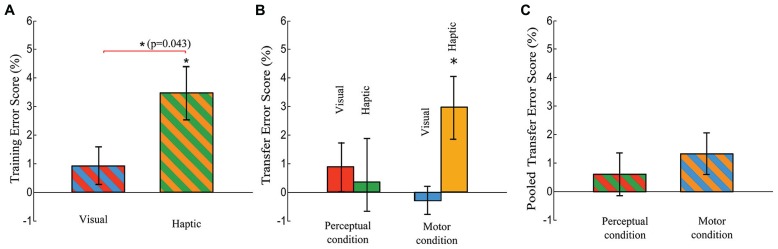
**Training Error Score and Transfer Error Score.** The Training Error Score is defined as a difference in error rate between the means of blocks 8 and 10 and block 9, and the Transfer Error score is defined as a difference in error rate between the means of blocks 12 and 14 and block 13. **(A)** Training Error Scores of the visual and haptic groups. There is a significant difference between the two groups (*t*-test, *p* = 0.043). **(B)** Transfer Error Score of the visual-perceptual, haptic-perceptual, visual-motor, and haptic-motor subgroups. **(C)** Pooled Transfer Error Score (averaged across the visual and haptic groups) in each condition. Error bars are ± 1 standard error of the mean. An asterisk on a line linking bars indicates a significant difference between two groups (subgroups) while an asterisk above a bar indicates a significant difference from zero.

While there was no difference in Training RT Scores across the visual and haptic groups, the difference in Training Error Scores reached significance [*t*(30) = 2.117, *p* = 0.043], as displayed in **Figure 6A**. Each Training Error Score quantifies the increase in error rate of pseudorandom block 9 over the average error rate of sequence blocks 8 and 10.

### TRANSFER RT SCORES AND TRANSFER ERROR SCORES

In the transfer phase (blocks 11 through 14) all participants responded to cues using a compatible mapping. The cues were delivered during the transfer phase such that the visual-perceptual and haptic-perceptual subgroups experienced cues that preserved the stimulus sequence across blocks 10 and 11, while the visual-motor and haptic-motor subgroups experienced cues that preserved the motor response sequence across blocks 10 and 11. Thus there were four Transfer RT Scores and four Training Error Scores computed, one each for each of the four subgroups.

One-tailed *t*-tests revealed that the Transfer RT Scores were significantly greater than zero in all the subgroups with the exception of the haptic-perceptual subgroup which was close as well (the haptic-perceptual subgroup: *p* = 0.068). Asterisks over the bars indicate the significant differences from zero in **Figure 5B**. These significant differences from zero imply that all the subgroups other than the haptic-perceptual subgroup utilized the advantage of their skills gained during the training phase in reducing RTs at sequence block 13.

Meanwhile, one-tailed *t-*tests revealed that the Transfer Error Scores were significantly greater than zero in only the haptic-motor subgroup: *t*(7) = 2.710, *p* = 0.006, as indicated in **Figure 6B**. The other three subgroups did not show significant differences from zero (the visual-perceptual: *p* = 0.346, the visual-motor: *p* = 0.551, the haptic-perceptual: *p* = 0.788)

Two-way ANOVAs across the four subgroups were used to test for differences among subgroups, with Stimulus and Condition as between-subject variables. For the Transfer RT Scores (the increase in the average RT of pseudorandom blocks 12 and 14 over the RT of sequence block 13), the main effect of Stimulus and Stimulus × Condition interaction were not significant (all *p* > 0.1, **Figure 5B**). However, ANOVA revealed a significant main effect of Condition [*F*(1,28) = 4.43, *MSE *= 0.001, *p* = 0.044, $\eta_{p}^{2}$ = 0.137], implying that the knowledge was transferred better in the motor condition than in the perceptual condition, as exhibited in **Figure 5C**.

For the Transfer Error Scores (the increase in the average error rate of pseudorandom blocks 12 and 14 over the error rate of sequence block 13), the ANOVA reported no significant main effects of Stimulus and Condition and no Stimulus × Condition interaction (see **Figures 6B,C**).

In sum, we had participants practice a SRT sequence task in which responses were cued either visually or haptically. Responses were prolonged when haptically cued, but the two groups exhibited parallel slopes of improvement during sequence repetition. Though both the visual and haptic groups showed a similar amount of sequence learning in terms of RTs, the haptic group gained more sequence learning than the visual group in terms of the number of errors made. We note that the SRT task resulted in a greater amount of motor learning than perceptual learning, irrespective of the stimuli (visual or haptic).

### AWARENESS

It is noteworthy that the awareness survey revealed that the sequence knowledge participants had gained during the SRT task was “implicit.” Also, the awareness survey showed that participants in the haptic group perceived more difficulty in performing the SRT task than participants in the visual group.

**Table 1** summarizes the results of the awareness survey. For analysis of Questions 2 and 3, inclusion, and exclusion scores were calculated for each participant and averaged per subgroup (see **Table 1**). However, since the participants were told to recall the sequence experienced in the training phase (the same sequence was presented regardless of Condition), we considered only the visual and haptic groups, which were distinguished strictly by Stimulus. One-tailed *t-*tests were used to compare the mean inclusion and exclusion scores (collapsed by modality as there were no reliable group differences) to chance level (0.33). This revealed that both inclusion [*t*(15) = 2.763, *p* = 0.007] and exclusion [*t*(15) = 3.201, *p* < 0.005] scores in the haptic group are reliably greater than chance. The visual group’s inclusion scores were greater than chance [*t*(15) = 3.844, *p* < 0.005] but the exclusion scores were not [*t*(15) = 1.069, *p* = 0.151], which would traditionally indicate explicit knowledge. However, paired-sample *t-*tests between inclusion and exclusion scores for the visual [*t*(15) = 1.438, *p* = 0.171] and haptic [*t*(15) = 0.115, *p* = 0.910] groups show that neither group’s means were significantly different, suggesting that participants did not recognize the sequence they experienced. The idea that such recognition should exhibit itself among participants with explicit knowledge is central to the application of the PDP in the SRT task along with the notion that comparing scores to chance level is not, by itself, an accurate indicator of awareness. As a significant difference was not observed in the visual group nor the haptic group, we could assert that sequence knowledge was largely implicit.

**Table 1:** A summary of awareness survey results.

		Visual-perceptual	Visual-motor	Haptic-perceptual	Haptic-motor
Question 1	Option 1	*n* = 0	*n* = 2	*n* = 1	*n* = 1
	Option 2	*n* = 0	n = 0	*n* = 0	*n* = 2
	Option 3	*n* = 3	*n* = 2	*n* = 2	*n* = 4
	Option 4	*n* = 5	*n* = 4	*n* = 5	*n* = 1
Question 2		0.458 ± 0.099	0.417 ± 0.126	0.469 ± 0.117	0.417 ± 0.204
Question 3		0.364 ± 0.133	0.375 ± 0.173	0.448 ± 0.133	0.427 ± 0.144
Question 4		*n* = 2	*n* = 2	*n* = 4	*n* = 2
Question 5		4.5 ± 0.534	4.625 ± 0.518	4.5 ± 0.534	4.875 ± 0.354
Question 6		3.0 ± 0.534	2.625 ± 0.744	4.0 ± 0.756	3.5 ± 1.195

*The rows for Questions 1 present how many participants chose each option. The row for Questions 4 presents how many participants chose the correct answer. The remaining rows present the averaged values by subgroup with standard deviations.*

In Question 4, all subgroups except for the haptic-perceptual subgroup had only two members recognize the correct sequence. Four members of the haptic-perceptual subgroup identified the correct sequence. One member of the visual-perceptual subgroup was not able to complete this question.

**Table 1** also presents participants’ average scores for Questions 5 and 6. For analysis of Questions 5 and 6, two-way ANOVAs were conducted with Stimulus and Condition as between-subject variables and Awareness Score as a within-subject variable. For Question 5 (how engaged participants were in the task), ANOVA revealed no significant main effects or interactions. For Question 6 (about the difficulty of the task), ANOVA reported a significant main effect of Stimulus [*F*(1,28) = 9.9, *MSE *= 7.031, *p* < 0.005, $\eta_{p}^{2}$ = 0.261], with the haptic group showing higher scores than the visual group. The main effect of Condition and interaction of Stimulus by Condition were not significant.

## DISCUSSION

We set out to determine whether haptic cues delivered to the fingertips would favor motor-based learning over perceptual learning, thinking that the effect would be even stronger with haptic cues than with visual cues. Willingham (1999) showed that sequence learning with visual cues favors motor learning over perceptual learning. Thus we expected a change in the cueing across the transition from incompatible to compatible mapping that preserved the sequence of motor responses would enable our participants to respond more quickly and with fewer errors than a change in cueing that preserved the sequence of stimuli. But further, we expected this effect to be stronger for a participant group that received haptic cues delivered to the fingers than a participant group that received visual cues. We hypothesized that cues delivered directly to the fingers would have a closer relationship to the responses produced by the fingers than cues delivered to the eyes. Roughly, we thought that haptic cues might engage “motor memory” by virtue of being delivered to the body site of the motor apparatus involved in responding to the cues.

### IMPLICIT LEARNING IN THE TRAINING PHASE

In general, participants responded more slowly to haptic cues than to visual cues. This result is consistent with Abrahamse et al. (2008), which reported that response times were noticeably longer with vibrotactile stimuli than with visual stimuli. These results are likely due to distinct processing of visual and haptic cues and perhaps distinct pathways between processing centers or centers that mediate learning (Cohen and Shoup, 1997; Keele et al., 2003; Goschke and Bolte, 2012). Another factor may be the longer pathway to the brain from the manual haptic receptors than from the retina. Nonetheless, the rates of RT improvement through blocks 2–8 were not significantly different between the visual and haptic groups (parallel downward slopes can be noted in **Figure 3**).

While the RTs for the haptic group were longer, the savings in RT enabled by the presence of the sequence once the sequence was learned (encapsulated in the Training RT Score) indicate a similar degree of learning during training between the visual and haptic groups. This contrasts with the result of Abrahamse et al. (2008) who showed that visual cues produce better sequence learning than vibrotactile cues. We attribute these differences to the greater salience of haptic cues delivered to the fingertips in the current study than the vibrotactile cues delivered to the proximal phalanx in Abrahamse et al. (2008).

The haptic group also demonstrated a significantly better Training Error Score than the visual group (*p* = 0.043, **Figure 6A**), indicating a stronger reliance on the presence of the sequence in the haptic group at the end of the training phase. Note that the lack of significant difference in Training RT Score between the visual and haptic groups indicates that the participants were not trading off error rate for RT performance. The difference in Training Error Score but lack of significant difference in Training RT Score may indicate that haptic cues favor response-response learning (Abrahamse et al., 2008) or that learning for our haptic group was encoded in the “what” of sequence execution and not so much in the “how”. We will discuss this distinction at greater length in light of the Transfer Result below.

### IMPLICIT LEARNING IN THE TRANSFER PHASE

Our central hypothesis, that haptic cues would favor motor learning even more than visual cues, was not supported by the differences in RT across our participant subgroups. There were no significant differences in the Transfer RT Score across the four subgroups (see **Figure 5B**). Only when the subgroups were pooled together into a motor group and a perceptual group, were differences in RT Score across condition significant (*p* = 0.044), as shown in **Figure 5C**.

It is curious that our visual-motor subgroup did not outperform our visual-perceptual subgroup, as the experimental conditions experienced by these subgroups were by design essentially equivalent to those found in (Willingham, 1999, Experiment 3), which established that sequence learning transfers better in the motor condition under visual cueing. Differences in our experiment design did exist, however; we used the index and ring fingers in each hand rather than the index and middle fingers of each hand. We would expect an elevated motor-based learning versus perceptual learning with the use of the index and ring fingers instead of the adjacent fingers, because it would facilitate the ability to discriminate the locations of responding fingers when coded in the egocentric space, a spatial frame that codes the locations of objects relative to part of the body. But our result does not duplicate Willingham’s result. Perhaps the design of our buttons (which required a throw of more than 5 mm) or other features of our arrangement are responsible for the difference. Our result is, however, consistent with his following study (Bischoff-Grethe et al., 2004). Bischoff-Grethe et al. (2004) also employed the same experimental protocol. This study reported that there was no significant difference between the perceptual and motor groups regardless of the extent of knowledge about sequences, although four fingers of one hand were used to respond to visual stimuli.

In terms of error rates, our central hypothesis did receive some support from our experiment. Participants in the haptic-motor subgroup made on average 3% fewer errors when the sequence was present in the transfer phase while the other three subgroups did not show a reduction in errors. That is, the haptic-motor subgroup realized a 3% increase in Transfer Error Score (significantly different from zero, *p* = 0.006) while the other three subgroups had Transfer Error Scores that were not significantly different from zero. Taken together, the support our central hypothesis received from the Transfer Error Scores and lack of support our central hypothesis received from the Transfer RT Scores may indicate that haptic cues favor only certain aspects of motor learning. That is, perhaps motor learning must be defined more narrowly in the context of sequence learning. It has been suggested that higher error rates indicate that sequence execution is influenced not so much by the stimuli but by the previously learned motor or movement patterns. In this sense, increased error rates at a pseudorandom block relative to surrounding sequence blocks could be interpreted as an indication that movement patterns were guided by the preceding movements rather than the cues. Evidently only the haptic-motor subgroup profited from the involvement of this type of association in learning (**Figure 6B**). This pattern of exhibiting sequence-specific knowledge in terms of accuracy has also been interpreted as participants knowing “what” to do for sequence execution. The lack of a comparable effect size in RT suggests that they cannot translate this into “how” to perform the sequence quickly (Hikosaka et al., 1999; Seidler et al., 2007). It is perhaps reasonable to assume that “what” and “where” is a kind of embodied knowledge, relying on motor memory or a motor program that is cued by reafferent proprioceptive stimuli, while “how” is more central, relying on stimuli collected from various external sources and assembled centrally into a pattern and program. It is possible that haptic cues are in fact more readily associated with a motor response in the sense of a motor program that governs positioning in the allocentric space, a spatial frame that codes the locations of objects relative to the environment. Note, however, that our current experiment did not test this hypothesis explicitly. The association is worth further exploration.

The lack of a significant interaction effect on Transfer Error Score, an indicator of whether haptic cues contribute differently than visual cues to the balance of motor and perceptual learning, might be due to the sample size. The partial eta squared ($\eta_{p}^{2}$) for the interaction effect was reported as 0.121. We found through a power analysis that 17 participants per subgroup would be needed to detect significance with power of 0.80 and alpha of 0.05. It may be that, with larger sample sizes, the Transfer Error Score interaction would have been significant. This should be explored in future studies.

The results of the awareness survey also indicated that the haptic group developed a stronger motor representation than the visual group. Four participants in the haptic-motor subgroup responded “Sometimes I wanted to respond before stimulus presentation,” which is greater than in the other subgroups.

In sum, we had participants practice a SRT task in which responses were cued either visually or haptically. Responses were prolonged when haptically cued, but the two groups exhibited parallel slopes of improvement during sequence repetition. Though both the visual and haptic groups showed a similar amount of sequence learning in terms of RTs, the haptic group gained more sequence learning than the visual group in terms of the number of errors made. We found that irrespective of the stimuli (visual or haptic) the SRT task leads to a greater amount of motor learning than perceptual learning. Moreover, transfer tests revealed that the haptic group acquired a stronger motor-based representation than the visual group in terms of the number of errors made.

## Conflict of Interest Statement

The authors declare that the research was conducted in the absence of any commercial or financial relationships that could be construed as a potential conflict of interest.
